# Analysis and Forecasting of Clock Product Deviation for BDS-3 PPP-B2b Based on the Normalized Time–Frequency Transform

**DOI:** 10.3390/s26175418

**Published:** 2026-08-27

**Authors:** Miao Wu, Jinyu Liu, Yifeng Liang, Yufei Yang, Qiuyang Luo, Haoke Zhan

**Affiliations:** 1 Naval University of Engineering, Wuhan 430033, China; 2 Beijing Satellite Navigation Center, Beijing 100094, China

**Keywords:** BDS-3, PPP-B2b, clock product deviation, normalized time–frequency transform, dynamic ridge, short-term prediction

## Abstract

**Highlights:**

**What are the main findings?**

- The normalized time–frequency transform (NTFT) dynamic ridge model reveals that the BDS-3 PPP-B2b clock product deviations (CPDs) contain three cross-satellite consistent time-varying periodic structures. These include the fundamental period, sub-harmonics, and envelope modulation components. The modeling reconstruction residual STD is reduced from 5.273 ns to 0.429 ns, corresponding to a 91.9% reduction relative to the fixed baseline.
- The proposed unified ridge envelope extrapolation (uREE) short-term forecast method extrapolates the amplitude envelope trend and phase evolution regularities into the forecast horizon. It reduces the RMS of the independent forecast dataset from 3.649 ns to 1.665 ns. It also decreases the systematic bias contribution from 83% to 18.5%.

**What are the implications of the main findings?**

- The research perspective on CPD is broadened from static bias estimation to time-varying periodic characterization. The results demonstrate that the fixed harmonic assumption does not hold for CPD. Conventional fixed harmonic models neglect the time-varying drift of amplitude and phase. This neglect is the primary source of systematic bias in existing forecast methods.
- The physics-guided uREE achieves a forecast accuracy improvement of over 57% for satellites with strong periodicity and outperforms all four evaluated benchmark models. It provides a generalizable methodological framework for interpretable forecasting of GNSS real-time clock bias.

**Abstract:**

Real-time BDS-3 PPP-B2b satellite clock products exhibit satellite-dependent clock product deviations (CPDs) relative to post-processed precise clocks, an important error source for real-time precise positioning and timing. Previous studies largely treat CPDs as static statistics or estimable parameters, leaving their nonstationary periodic structure and prediction potential unexplored. A hierarchical analytical framework based on the normalized time–frequency transform (NTFT) is therefore established, whose dynamic ridge model tracks the joint time-varying trajectories of amplitude, frequency, and phase without period priors. The modeling residual standard deviation is reduced from 5.273 ns to 0.429 ns, and three cross-satellite common components are identified. Building on this structure, a unified ridge envelope extrapolation (uREE) method converts the envelope trend and phase evolution into short-term forecasts through three satellite adaptive modes. On an independent single-day test, uREE reduces the overall RMS from 3.649 ns to 1.665 ns, corresponding to a 54.4% improvement, and outperforms all four benchmark models. Bias–dispersion decomposition reveals that this gain stems from the mitigation of systematic bias (whose contribution falls from above 83% to 18.5%) rather than from random noise suppression. These results shift CPD research from static bias estimation to physically interpretable time-varying modeling and forecasting.

## 1. Introduction

BDS-3 PPP-B2b broadcasts real-time precise orbit, clock, and code bias corrections via geostationary Earth orbit (GEO) satellites, enabling real-time precise point positioning (PPP) and timing services without reliance on terrestrial communication networks [1,2,3,4,5]. Such services support applications that demand continuous, high-accuracy spatiotemporal information, including ocean monitoring, disaster early warning, and intelligent transportation. Because the estimation conditions, models, and processing strategies of real-time products differ from those of post-processed precise products, satellite-dependent CPDs of nanosecond magnitude commonly exist between the two. If these deviations are not adequately absorbed, they may propagate into positioning and timing solutions.

Existing studies have addressed the bias characteristics of the BeiDou system from the perspectives of pseudorange biases, time group delays (TGDs), and inter-frequency clock biases. Liu et al. [6] reported inter-satellite heterogeneity in PPP-B2b clock offsets. Tian et al. [7] and Zhang et al. [8] investigated BDS pseudorange biases and TGD errors, respectively. Wang et al. [9], Zhao et al. [10], Lu et al. [11], and Li et al. [12] examined the estimation of differential code biases, constant clock biases, and inter-frequency clock biases. However, these studies largely treat CPD-related biases as statistics or estimable parameters. Their internal time-varying periodic structure and potential value for short-term prediction remain insufficiently investigated.

Regarding GNSS satellite clock periodicity, Senior et al. [13] revealed periodic terms and seasonal amplitude variations in GPS satellite clocks. Their results demonstrate that satellite clock biases are not static parameters but complex signals with time-varying characteristics. Montenbruck et al. [14], Steigenberger and Montenbruck [15] and Steigenberger et al. [16] showed that solar radiation pressure modeling errors may propagate into orbit and clock products. Chen et al. [17] further identified satellite-dependent nonlinear discrepancies among clock products from different analysis centers. For BDS, Zhao et al. [18] employed the short-time Fourier transform (STFT) to quantify the time-varying amplitude and frequency of the dominant periodic term in clock offsets, and linked the periodic power to the β-angle and eclipse seasons. However, the aforementioned studies address the satellite clock offset itself rather than the deviation between real-time and post-processed products. The periodic characteristics of BDS-3 PPP-B2b real-time CPD remain insufficiently investigated.

For period extraction, the discrete Fourier transform (FFT) fits the entire series with fixed parameters and cannot capture temporal evolution. The short-time Fourier transform (STFT) alleviates this limitation with localized spectra, yet its fixed window length entails a time–frequency resolution trade-off and provides no ridge continuity constraint. Empirical mode decomposition (EMD) is prone to mode mixing. Variational mode decomposition (VMD) requires a preassigned number of modes. The NTFT extracts local complex coefficients at a prescribed frequency via a normalized Gaussian kernel, and was developed for long-term polar motion prediction [19] and short-term tidal level prediction [20]. This ensures normalized and unbiased extraction. However, the periods of CPD are unknown a priori and may drift over time. Therefore, direct application of the NTFT cannot achieve adaptive period tracking. To overcome this limitation, we construct an NTFT dynamic ridge model. The model screens candidate periods using a periodogram and tracks the optimal ridge through dynamic programming, following established ridge extraction techniques [21,22,23]. It thereby adaptively extracts the joint time-varying trajectories of amplitude, frequency, and phase without prior frequency knowledge.

For prediction, Lv et al. [24] identified insufficient modeling of periodic terms as a key bottleneck limiting the accuracy of clock bias prediction. Ge et al. [25] systematically evaluated the polynomial, grey, and ARIMA models on GNSS precise clock products and found that their accuracy strongly depends on the clock type and the lengths of the fitting and prediction arcs. Zhao et al. [18] addressed the periodicity of this bottleneck by constructing a time-frequency analysis model based on the STFT, which compensates the time-varying periodic terms of BDS clocks and improves the prediction accuracy. Data-driven predictors for BDS-3 clock bias have also emerged. Representative works include the EWT-LSTM ensemble model [26] and a low sampling estimation for real-time service [27]. However, the STFT-based compensation relies on a fixed window length and targets the clock offset itself, whereas the data-driven methods are essentially black-box mappings that depend on large training samples and offer limited physical interpretability. In addition, the NTFT provides only local time–frequency states within the modeling interval. Direct extrapolation of these states may introduce systematic deviations arising from envelope slope and phase drift mismatch. We therefore propose the uREE method. It extends the envelope trend and phase evolution estimated at the end of the modeling interval into the prediction interval. We evaluate it against fixed baseline, FFT-anchored, k-nearest neighbor (KNN), and radial basis function kernel ridge regression (RBF-KRR) predictors under identical experimental conditions.

The differences between the proposed framework and relevant counterpart methods are summarized in Table 1.

The main contributions of this study are summarized as follows:

1. An NTFT-based dynamic ridge model is established, in which periodogram screening and dynamic programming ridge tracking jointly extract the time-varying trajectories of amplitude, frequency, and phase without period priors, reducing the modeling residual standard deviation from 5.273 ns to 0.429 ns.
2. Three cross-satellite common components of the CPD are identified: a fundamental period consistent with 14 sidereal days, fractional sub-harmonics, and an envelope modulation governed by the β-angle, which form a coupled hierarchy rather than independent spectral lines.
3. A unified ridge envelope extrapolation (uREE) method is proposed to extend the envelope trend and phase evolution into the prediction horizon through three satellite adaptive modes, reducing the one-day forecast RMS from 3.649 ns to 1.665 ns (54.4% improvement) and outperforming all four benchmark models under identical experimental conditions.
4. Bias–dispersion decomposition attributes the forecast gain to systematic bias mitigation, whose contribution falls from above 83% to 18.5%, rather than to random noise suppression.

## 2. Methodology

**Figure 1:**
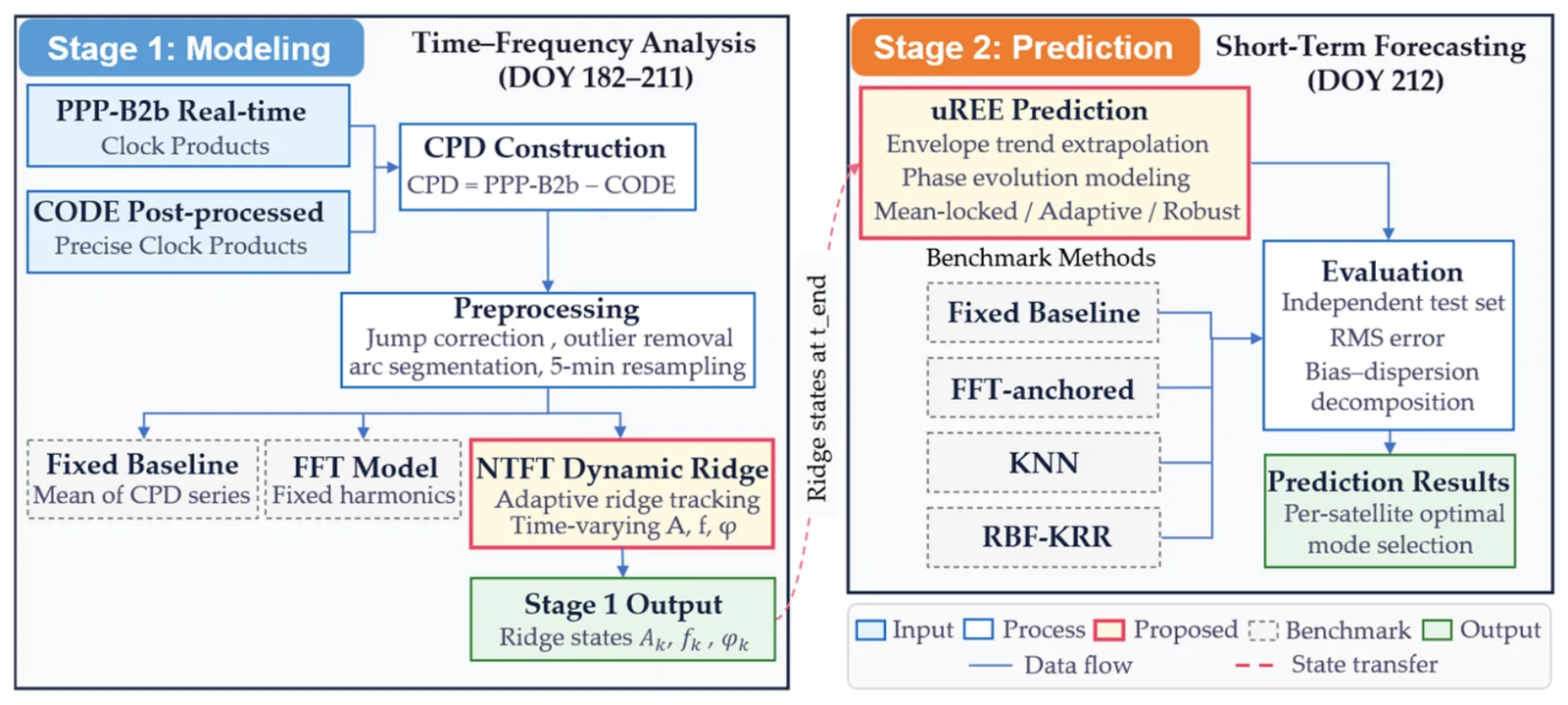
Overview of the proposed framework.

The overall workflow of the proposed framework is illustrated in Figure 1.

The framework consists of two stages. Stage 1 constructs the CPD series from the PPP-B2b real-time and CODE post-processed clock products and characterizes its nonstationary periodic structure through the NTFT dynamic ridge model. Stage 2 extends the ridge states estimated at the end of the modeling interval into the prediction horizon via the proposed uREE method and evaluates the forecast against four benchmark strategies. The remainder of this section details each stage in turn.

### 2.1. Data and Definition of CPD

The experiment employs 30 days of BeiDou 3 Precise Point Positioning B2b (BDS-3 PPP-B2b) real-time clock products spanning day of year (DOY) 182–211 of 2025. Post-processed precise clocks from the Center for Orbit Determination in Europe (CODE) serve as the reference for constructing CPD series, while DOY 212 is reserved as an independent prediction set. All data are aligned by satellite identifier, corrected for clock jumps, cleaned of outliers, and arc-segmented, and are then uniformly resampled onto a regular 5 min grid.

The satellite-dependent CPD at epoch $t_{i}$ is defined as

(1) $$y(t_{i})=t_{clk}^{B2b}(t_{i})-t_{clk}^{Fin}(t_{i})$$

where $t_{clk}^{B2b}(t_{i})$ and $t_{clk}^{Fin}(t_{i})$ denote the PPP-B2b real-time clock offset and the CODE post-processed precise clock offset at epoch $t_{i}$, respectively. To facilitate the subsequent decomposition, the CPD sequence is further expressed as [13]

(2) $$y(t_{i})=b_{0}+\tau (t)+\sum_{k=1}^{K}s_{k}(t)+\varepsilon (t)$$

where $b_{0}$ is a stable constant bias; τ(t) is a slowly varying trend term; $s_{k}(t)$ is the kth time-varying periodic component; ε(t) is random noise; and K is the number of periodic components. This expression is an analytical decomposition and does not prescribe the physical origins of the individual components a priori.

### 2.2. Time–Frequency Analysis Framework

The analysis proceeds in two stages. First, a fixed baseline, an FFT model, and an NTFT dynamic ridge model are established sequentially to determine a constant bias lower bound, to construct a stationary periodic reference, and to achieve nonstationary time-varying modeling, respectively. Second, the local time–frequency state extracted by NTFT at the end of the modeling interval is fed into uREE for short-term extrapolation and is compared with four strategies under identical conditions.

#### 2.2.1. Fixed Baseline and FFT Model

For *N* epochs in the modeling interval, the fixed baseline is taken as the sample mean of the CPD series.

(3) $${\hat{y}}_{fixed}=\frac{1}{N}\sum_{i=1}^{N}y(t_{i})$$

The fixed baseline does not explain trends, periods, or phase variations, yet it constitutes a necessary prediction benchmark. Only when a more complex model stably outperforms this benchmark on independent prediction tests can the introduced time-varying structure be regarded as having practical extrapolative value.

After removal of the slowly varying trend, the FFT model fits a superposition of sinusoids with fixed amplitudes and fixed phases [13]

(4) $$\begin{array}{c} {\hat{y}}_{FFT}(t)=\tau_{poly}(t)+\sum_{k=1}^{K}A_{k}\sin(\omega_{k}+\phi_{k}),\;K\leq 3\\ \tau_{poly}(t)=c_{0}+c_{1}t+c_{2}t^{2} \end{array}$$

where $A_{k}$ and $\phi_{k}$ are the fixed amplitude and phase of the kth periodic component, respectively. The frequencies are first identified from the FFT periodogram, after which the linear amplitudes and phases are estimated by least squares on the original epochs for each candidate frequency. This model is used to test whether CPD exhibits dominant periods in an average sense; its assumption is that each period maintains fixed frequency, amplitude, and phase over the entire interval.

#### 2.2.2. NTFT Dynamic Ridge Model

To overcome the stationarity limitation of the FFT model, the NTFT is adopted for time–frequency analysis. The NTFT performs a localized complex projection onto the target frequency via a normalized Gaussian window, and the complex coefficients read along the optimal ridge directly reconstruct the time-varying periodic components.

For a discrete signal y[n](n=0,1,…,N−1) with sampling rate $f_{s}$, the NTFT complex coefficient at analysis frequency $f_{k}=1/T_{k}$ is defined as [19,20,28]

(5) $$C(f_{k},n)=\sum_{l}y[n+l]{\tilde{h}}_{f}[l]\exp(j\frac{2\pi f_{k}l}{f_{s}})$$

where the discrete Gaussian window is

(6) $${\tilde{h}}_{f}[l]=\frac{1}{s_{f}}\exp(-\frac{l^{2}}{2\sigma_{f}^{2}})\begin{array}{cc} , & s_{f} \end{array}=\sum_{l=-L}^{L}\exp(-\frac{l^{2}}{2\sigma_{f}^{2}})$$

where $s_{f}$ is the discrete normalization factor ensuring $\sum_{l}{\tilde{h}}_{f}[l]=1$. The standard deviation of the Gaussian envelope $\sigma_{f}$ controls the effective width of the time-domain analysis window, and the modulation term exp(⋅) implements the localized projection at the target frequency $f_{k}$.

The NTFT possesses two fundamental properties: normalization and unbiased extraction. The normalization property ensures that the amplitudes of different frequency components are directly comparable. The Gaussian window response in the frequency domain remains approximately Gaussian, peaking at the target frequency and decaying rapidly with frequency deviation.

(7) $${\hat{\tilde{h}}}_{k}(\xi )\propto \exp[-\frac{\sigma_{\xi }^{2}{\left(\xi -\xi_{k}\right)}^{2}}{2}]$$

where $\xi_{k}=f_{k}/f_{s}$ is the normalized analysis frequency and $\sigma_{\xi }\propto 1/\sigma_{f}$ is the frequency domain bandwidth.

Regarding the unbiased extraction property, consider a discrete single-component harmonic signal:

(8) $$y[n]=A\cos(\frac{2\pi f_{0}n}{f_{s}}+\phi )$$

where A is the amplitude, $f_{0}$ is the true frequency, and ϕ is the initial phase. When the analysis frequency $f_{k}$ approaches $f_{0}$, the NTFT complex coefficient becomes:

(9) $$C(f_{k},n)=A\cdot {\hat{H}}_{std}(0)\cdot \exp[j\phi (n)]\approx A\exp[j\phi (n)]$$

where $\phi (n)=2\pi f_{0}n/f_{s}+\phi_{0}$ is the local instantaneous phase and ${\hat{H}}_{std}(\cdot )$ denotes the frequency response of the Gaussian window after time-domain integration normalization, with ${\hat{H}}_{std}(0)=1$. Consequently, the complex coefficient at the ridge directly equals the local analytic representation of the signal: its modulus yields the instantaneous amplitude and its argument yields the instantaneous phase, without requiring additional fitting.

The unbiased extraction property constitutes the theoretical foundation of passive reconstruction. The term “passive” signifies that the complex coefficients are read directly from the optimal ridge to recover the local analytic features of the time-varying component, thereby distinguishing the NTFT from the CWT, whose amplitude depends on scale normalization.

Based on the above principles, the NTFT dynamic ridge modeling of CPD proceeds as follows.

1. Trend separation.

A robust smoothing spline estimated by iteratively reweighted least squares (IRLS) is used to obtain the slowly varying trend, thereby reducing the influence of outliers and local discontinuities on low-frequency components.

(10) $$\begin{array}{c} \tau^{*}=\arg\underset{\tau }{\min}\sum_{i}w_{i}[y(t_{i})-\tau (t_{i})]2+\lambda {\int \left[\ddot{\tau }(t)\right]}^{2}dt\\ w_{i}=\frac{1}{\max(1,\frac{\left|r_{i}\right|}{2.5\sigma_{MAD}})},u_{i}=y(t_{i})-\tau (t_{i}) \end{array}$$

where $y(t_{i})$ is the raw CPD value at epoch $t_{i}$; $\tau (t_{i})$ is the trend to be estimated; λ is the smoothing parameter controlling trend curvature; $w_{i}$ is the robust weight at epoch i; $\sigma_{MAD}$ is the median absolute deviation (MAD) of the residuals, MAD is computed by first taking the median of the residuals as the center, then taking the median of the absolute deviations of each residual from this median; and $u_{i}$ is the current iteration residual.

IRLS down weights epochs with abnormally large residuals. The detrended residual then serves as the input for candidate period screening and ridge tracking.

2. Candidate period screening.

Candidate periods are ranked by a joint score combining periodogram power, ridge-averaged amplitude, and stability:

(11) $$\begin{array}{c} Score(T)=\frac{P(T)}{P_{\max}}[0.45+0.55Stab(T)]\max(\bar{A}(T),10^{-6})\\ Stab(T)=\frac{1}{1+CV_{A}(T)+0.35\sigma_{\Delta \phi }(T)} \end{array}$$

where P(T) is the periodogram power at period T; $P_{\max}$ is the maximum power over the full frequency band; $\bar{A}(T)$ is the time-averaged ridge amplitude at period T; Stab(T) is the stability index ranging over (0,1]; $CV_{A}(T)$ is the coefficient of variation (*CV*) of the ridge amplitude; and $\sigma_{\Delta \phi }(T)$ is the standard deviation of the first-order phase difference, penalizing envelope fluctuation and frequent phase jumps. The top K candidate periods are selected in descending order of composite score. Candidate periods are searched over 24–480 h, with the Welch periodogram estimated using a Hann window of 168 h and 50% overlap. Because the three indicators carry different units (normalized power, ns, dimensionless), each is min–max normalized to [0, 1] over the candidate set before linear combination. *S*(*T*) is therefore dimensionless, and the weights represent relative importance only.

3. Candidate period screening.

A 17-point frequency grid is established within the ±12% neighborhood of each candidate period $T_{k}$, and dynamic programming is employed to identify the ridge that maximizes cumulative energy while ensuring smooth variation between adjacent epochs:

(12) $$D(q,n)=\underset{p\in \{1,\ldots ,17\}}{\max}\{D(p,n-1)+\tilde{A}(q,n)-\eta \rho (n){\left(q-p\right)}^{2}\}$$

where D(q,n) is the maximum cumulative score at epoch n and grid point q; $\tilde{A}(q,n)$ is the normalized instantaneous amplitude at grid point q and epoch n; η is the global jump penalty coefficient; ρ(n) is the time-varying penalty weight; and ${\left(q-p\right)}^{2}$ quantifies the grid point jump between adjacent epochs. The penalty term $\eta \rho (n){\left(q-p\right)}^{2}$ suppresses ridge frequency jitter. Specifically, η was tuned by a grid search on the training arc: candidate values from 0.05 to 0.5 in steps of 0.05 were tested, and the smallest value that suppressed visible ridge jitter without attenuating genuine frequency drift was adopted (η=0.2 in this study). The weight ρ(n) is inversely proportional to the local ridge amplitude and normalized to the interval [0.5, 1], so that epochs with weaker ridge energy, where tracking is less reliable, receive a stronger smoothness penalty. After completing the recursion across all 17 grid points at each epoch, backtracking from the final epoch yields the optimal grid point sequence $q_{n}^{*}$, constituting the dynamic ridge of the candidate component.

4. Passive reconstruction.

For the kth periodic component, the NTFT complex coefficient is read from the optimal ridge $q_{n}^{*}$ and the real-valued signal is reconstructed as:

(13) s^k[n]=2Re{Ckridge[n]}

where $C_{k}^{ridge}[n]$ is the NTFT complex coefficient of the kth periodic component at the ridge grid point $q_{n}^{*}$ and 2Re{⋅} yields the real-valued reconstruction of the component at epoch n.

To absorb the residual slowly varying components beyond the ridge-extracted periodic terms, moving average windows of 6, 12, 24, 48, and 72 h are applied to construct low-frequency corrections, and the window minimizing the post-correction residual standard deviation on the modeling set is selected:

(14) $$r_{win}(t)=MA_{w^{*}}[y(t)-{\sum_{k}\hat{s}}_{k}(t)]$$

where $MA_{w^{*}}(\cdot )$ denotes the moving average low-pass operator with window width w hours, retaining only components with time scales exceeding w hours; the optimal window $w^{*}$ is determined by minimizing the post-correction residual standard deviation.

The NTFT dynamic ridge model is therefore written as:

(15) $${\hat{y}}_{NTFT}(t)=\tau^{*}(t)+\sum_{k=1}^{K}{\hat{s}}_{k}(t)+r_{win}(t),\;K\leq 3$$

where the three terms account for the slowly varying trend, the time-varying periodic structures, and the residual low-frequency component. The key advantage of passive reconstruction lies in its complete preservation of the time-varying amplitude envelope and the nonstationary phase drift.

### 2.3. Short-Term Prediction Methods

#### 2.3.1. Unified Ridge Envelope Extrapolation (uREE)

uREE extends the local time–frequency states extracted by the NTFT during the modeling stage to the prediction interval. Given that the periodic intensity and stability vary across satellites, three candidate modes—mean-locked, adaptive, and robust—are designed, and the optimal mode for each satellite is selected via rolling hold-out validation.

1. Mean-locked mode

This mode is suitable for satellites with clearly defined periodic structure and a relatively stable mean level, corresponding to the high-amplitude stable type classified in Section 3.2. The prediction is centered on the fixed mean of the modeling segment, superimposed with time-varying periodic components:

(16) $${\tilde{y}}_{ml}(t)=\bar{y}+\sum_{k\in M}A_{k}(t)\cos[\omega_{k}t+\phi_{k}(t)]$$

where the amplitude $A_{k}(t)$ is a weighted combination of recent and global estimates with weights 0.65 and 0.35, subject to the constraint $A_{k}(t)\leq A_{k}^{global}+\sigma_{A_{k}}$. The phase $\phi_{k}(t)$ is determined by least-squares fitting $a\sin\omega_{k}t+b\cos\omega_{k}t$ over the final H h of the modeling segment, yielding $\phi_{k}(t)=\arctan2(b,a)$.

2. Adaptive mode

This mode applies a convex combination of the mean-locked prediction and the fixed baseline, intended for satellites with weak periodic evidence or unstable local states, corresponding to the medium amplitude universal type in Section 3.2:

(17) $${\tilde{y}}_{ad}(t)=w\cdot {\tilde{y}}_{ml}(t)+(1-w)\cdot \bar{y}$$

where the weight w∈[0,1] is determined by rolling hold-out validation on the modeling set, automatically degenerating to the fixed baseline when local periodic information is unstable.

3. Robust mode

This mode relaxes the frequency constraint and directly performs linear extrapolation of the envelope $E_{k}(t)$ and phase $\Phi_{k}(t)$ derived from the NTFT ridge complex coefficients, allowing the recent frequency to deviate from the global average. It is suited for satellites with strong periodic structure but local frequency drift, corresponding to the low-stability anomalous type in Section 3.2:

(18) $$\begin{array}{c} E_{k}(t)\approx a_{k0}+a_{k1}(t-t_{0})\\ \Phi_{k}(t)\approx \phi_{k0}+\omega_{k}^{recent}(t-t_{0}) \end{array}$$

where $t_{0}$ is the terminal epoch of the recent window and $\omega_{k}^{recent}$ is the angular frequency obtained from the recent phase slope. The angular frequency used for extrapolation is a weighted combination of recent and global estimates:

(19) $$\omega_{k}=0.5\omega_{k}^{recent}+0.5\omega_{k}^{global}$$

The amplitude extrapolation $A_{k}(t)$ supports three strategies—linear continuation, linear plus global weighting, and terminal value hold—which are selected via cross-validation, subject to the upper bound $A_{k}(t)\leq \max(A_{k}^{global}+\sigma_{A_{k}},E_{k}(t_{0}))$.

The prediction is therefore expressed as:

(20) $${\tilde{y}}_{rb}(t)=\bar{y}+w\sum_{k\in M}A_{k}(t)\cos[\omega_{k}t+\Phi_{k}(t)]$$

The three modes are evaluated by four-fold rolling hold-out validation, and satellite-level selection is performed using a composite score that accounts for the average error, the most recent fold error, inter-fold stability, and phase risk:

(21) $$S=\bar{r}+0.5r_{4}+0.5\sigma (r_{i})+\lambda \varsigma$$

where $\bar{r}$ is the four-fold average error; $r_{4}$ is the RMS of the final fold; $\sigma (r_{i})$ is the standard deviation of the four-fold errors; ς is the phase instability; and λ is the corresponding penalty coefficient. The mode with the lowest score is assigned as the optimal prediction mode for the corresponding satellite, thereby achieving satellite-level adaptation.

#### 2.3.2. Benchmark Methods

Four benchmark strategies are configured to evaluate the gains of uREE over constant, stationary harmonic, and lightweight data-driven approaches.

1. The fixed baseline uses the mean of the modeling segment.
2. FFT-anchored method: A fixed harmonic variant of the spectral analysis model (SAM) widely used in ultra rapid clock prediction [18,25]. In standard SAM, the polynomial trend captures the dominant clock drift; because the CPD is a differenced series in which this drift is common mode and cancels, the trend degenerates to a constant-level correction, leaving fixed harmonic extrapolation as the effective forecasting content. This baseline extrapolates the global Fourier harmonics and corrects the level by the termina segment mean.
3. KNN: The feature vector comprises multi-scale lagged CPD values at lags of 1, 6, 12, 24, 48, and 72 h, together with the diurnal phase encoded as the sine and cosine of the hour of day. Regression is performed by inverse distance weighting over the nearest neighbors in the modeling set. The neighbor count is selected by exhaustively evaluating all candidates in {3, 5, 7, 9, 11} via cross-validation on the modeling set, and the candidate with the lowest validation RMS is adopted.
4. RBF-KRR: The ridge coefficient is searched over a logarithmic grid from 10^−4^ to 10^2^ in half-decade steps (13 values), and the RBF kernel bandwidth over a logarithmic grid from 10^−3^ to 10^1^ in half-decade steps (9 values). Both hyperparameters are jointly evaluated over the full 117-point grid, and the pair minimizing the validation RMS is selected, with no early stopping applied.

All models employ identical per-satellite data partitioning and prediction starting points, and neither preprocessing nor hyperparameter selection has access to the DOY 212 test data.

## 3. Experimental Results

### 3.1. Time–Frequency Characterization of CPD

This section conducts CPD modeling analysis based on NTFT spectrograms, identifies periodic components with stable time–frequency support and potential physical interpretation, and discusses their distributional differences among satellites.

**Figure 2:**
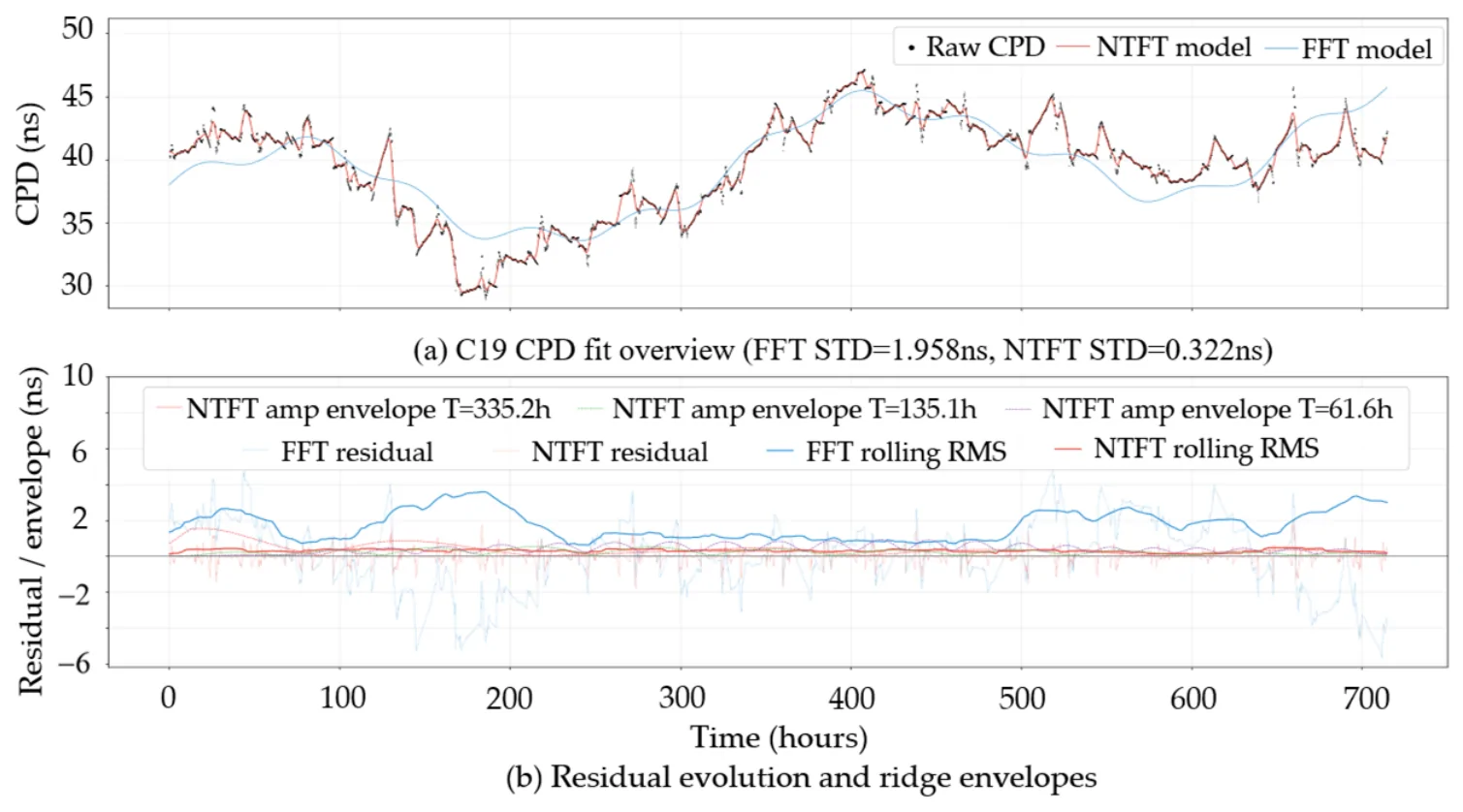
Schematic of time-domain fitting and residuals.

**Figure 3:**
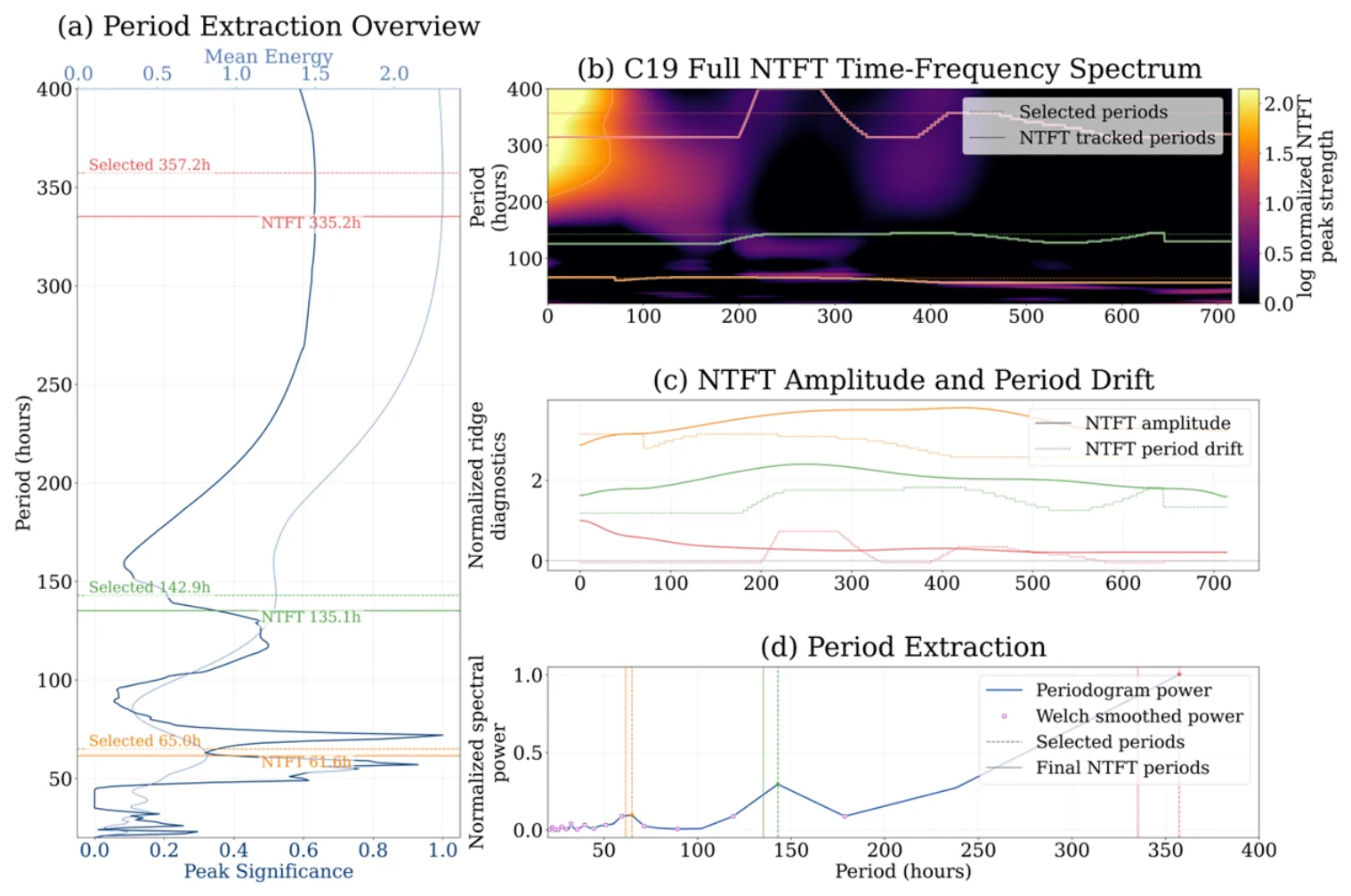
NTFT spectrogram and period support relations.

**Figure 4:**
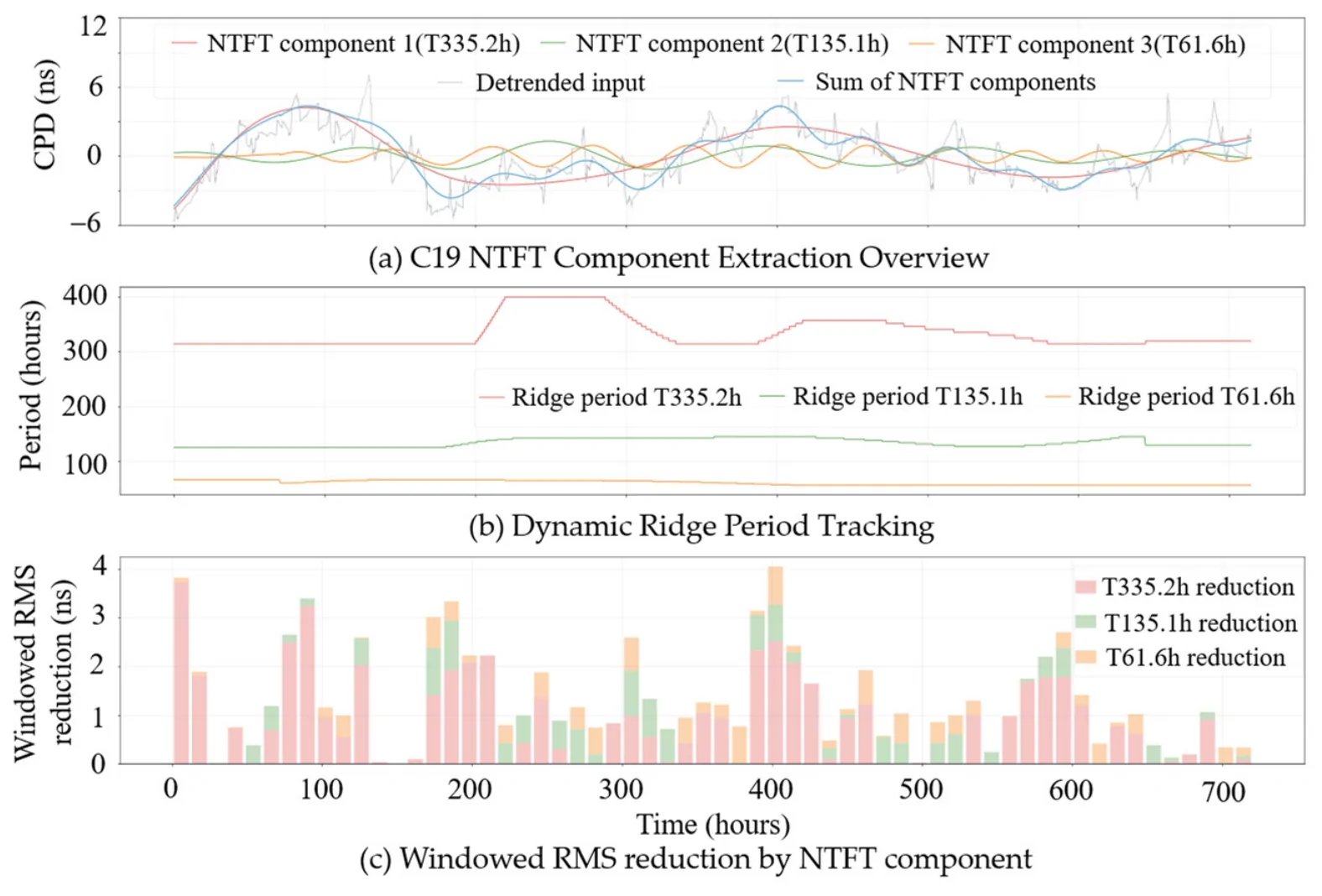
Dynamic ridge extraction and progressive residual reduction.

Taking satellite C19 as an example, Figure 2 compares the time-domain fit and residuals of NTFT reconstruction with those of the FFT model; Figure 3 presents the NTFT spectrogram of C19 and the associated period support; and Figure 4 shows the progressive residual reduction during dynamic ridge extraction.

As shown in Figure 2, the NTFT reconstruction accurately reproduces the temporal locations of peaks and troughs and preserves envelope undulations. Residuals concentrate within approximately ±1 ns and exhibit no evident systematic trend.

Figure 3 further reveals the nonstationarity of the CPD. The 335.2 h fundamental period ridge persists throughout the entire observation span with continuous energy, yet the ridge amplitude exhibits discernible fluctuations and the period trajectory undergoes slight drift. The Welch power spectral peak and the NTFT-detected period reside in a neighboring frequency band but exhibit an identifiable offset. Because the Welch spectrum is predicated on global stationarity, its spectral peak reflects the mean frequency position, whereas the NTFT ridge tracks the instantaneous frequency trajectory. When frequency drift or amplitude modulation is present, the two quantities diverge systematically. Therefore, this offset itself constitutes direct evidence of the nonstationary character of the CPD.

The instantaneous periods of the three ridge components fluctuate slowly around their nominal values, and the windowed RMS decreases progressively as each component is incorporated, ultimately stabilizing at approximately 0.3 ns (Figure 4). This indicates that the selected components account for the majority of the reproducible structure within the C19 modeling segment; the residual error may still contain measurement noise, unmodeled systematic terms, and preprocessing artifacts.

Cross-satellite statistics show that the mean modeling residual standard deviations for the fixed baseline, FFT, and NTFT dynamic ridge models are 5.273, 2.637, and 0.429 ns, respectively. The NTFT achieves a 91.9% reduction relative to the fixed baseline, demonstrating that local time–frequency parameters substantially improve in-sample reconstruction capability.

**Figure 5:**
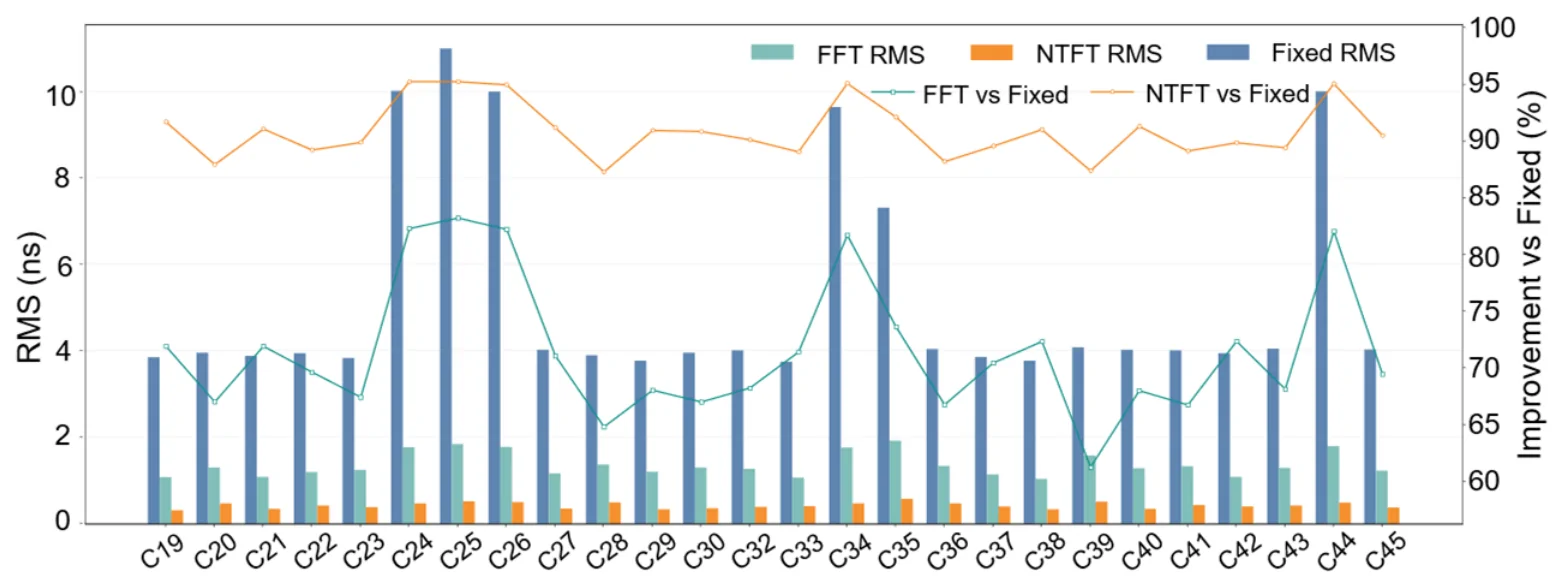
Per-satellite comparison of modeling residual RMS.

The accuracy advantage of the NTFT is consistent across satellites (Figure 5) and independent of specific satellites or orbit types, indicating that the time-varying periodic structures in the CPD are systematic components shared by the majority of satellites, although their amplitudes and stability vary among individual satellites. The residual RMS of satellites C24–C26, C34, and C44 under the fixed baseline exceeds 9 ns, substantially higher than that of the remaining satellites; the NTFT dynamic ridge model reduces their residuals to approximately 0.5 ns.

### 3.2. Identification of Periodic Components and Physical Relevance

The NTFT ridge yields the local instantaneous period, whereas the FFT spectral peak provides the global average period. Table 2 summarizes the three categories of periodic components that exhibit cross-satellite clustering in the dynamic ridge analysis.

**Figure 6:**
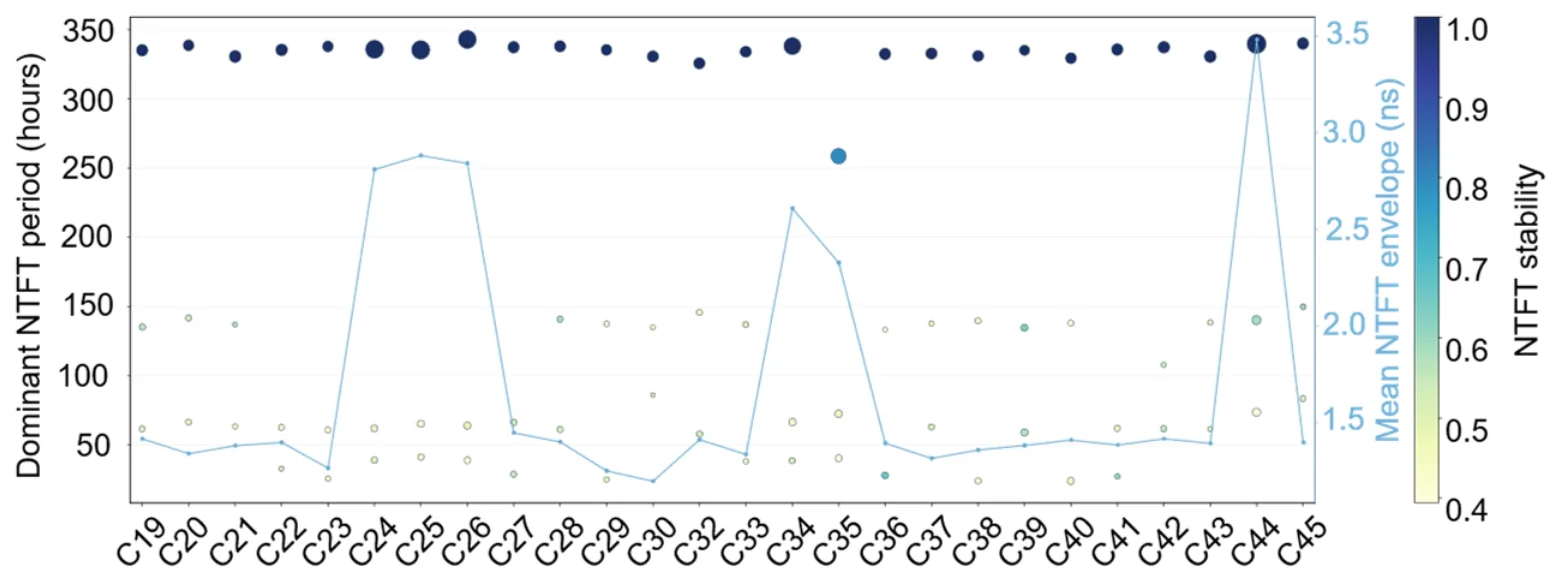
Distribution of CPD periodic structures.

Although the three periodic components exhibit cross-satellite consistency, their amplitudes and stability display significant inter-satellite variability. Figure 6 presents the period amplitude distribution and stability of the CPD periodic components across all satellites. The fundamental period and sub-harmonics are highly clustered among satellites, and the amplitude is positively correlated with stability.

Bubble size represents periodic intensity, color denotes stability, and the solid line indicates the mean envelope. Based on the above differences, the satellites can be classified into three categories, which correspond one-to-one to the three candidate modes of uREE (Section 2.3.1): the high-amplitude stable type is served by the mean-locked mode, the medium-amplitude universal type by the adaptive mode, and the low-stability anomalous type by the robust mode.

- High-amplitude stable type: C24–C26, C34, and C44, whose fundamental period amplitudes exceed 5 ns, with clearly defined periodic structure and favorable stability.
- Medium-amplitude universal type: comprising the majority of satellites, with amplitudes in the range of 2.1–3.0 ns and relatively stable periodic structure; these represent the broadest coverage of the uREE forecast model.
- Low-stability anomalous type: represented by C35, characterized by large amplitude but poor stability, potentially affected by non-periodic disturbances such as onboard clock frequency modulation perturbations and orbital maneuvers, resulting in low signal-to-noise ratio for period extraction.

#### 3.2.1. Fundamental Period: Association with 14 Sidereal Days

Excluding C35, the fundamental periods of the remaining 25 satellites yield a mean of 335.0 h with a standard deviation of 3.8 h, closely matching 14 sidereal days (335.08 h). The twice ground track repeat periods of individual satellites range from approximately 47.6 to 507.2 h, while the IGSO satellites exhibit a twice repeat period of approximately 47.6 h yet retain fundamental periods of approximately 329–335 h. The results do not support a unified explanation of this dominant component in terms of a simple single-satellite orbital repeat period. This component may be associated with 14-sidereal-day periodic effects in the Earth-fixed frame or with a combination of multiple periodic processes. This fundamental component anchors the CPD periodic hierarchy, and its stability determines how the lower order fractional components should be interpreted, as examined in the following subsection.

#### 3.2.2. Sub-Harmonic Components: Association with Fundamental Period Harmonics

The sub-harmonic components exhibit a bimodal distribution (Table 1): a component at approximately 133–150 h appears in 16 satellites, consistent with 2/5 of the fundamental period (134.0 h); a component at approximately 57–86 h appears in 21 satellites, consistent with the 1/5 (67.0 h) to 1/4 (83.8 h) range of the fundamental period. Both components overlap with integer multiples of the MEO orbital period (approximately 12.9 h), precluding discrimination between orbital harmonic and fractional harmonic interpretations on MEO satellites alone. The IGSO satellites provide discriminating evidence: if driven by the orbital period (approximately 23.8 h), IGSO satellites should exhibit components at approximately 48 h (2×) or 71 h (3×), yet none of the three IGSO satellites display such components, which does not support the orbital harmonic interpretation. Additionally, a small number of satellites exhibit short period components at approximately 24 h (7 satellites) and 33–41 h (7 satellites), approaching one sidereal day and 1/10 to 1/8 of the fundamental period, respectively. Overall, the sub-harmonics are more likely attributable to fractional harmonics of the fundamental period. These fractional components are not fixed spectral lines superimposed on the fundamental component; their energy fluctuates in step with the amplitude evolution of the fundamental ridge, which leads to the envelope modulation analysis below.

#### 3.2.3. Envelope Modulation Components: Association with β-Angle Modulation

The β-angle is defined as the angle between the Sun direction and the normal vector of the satellite orbital plane, characterizing the solar illumination geometry on the orbital plane. The β-angle drifts slowly as the orbital plane processes in the inertial frame, with its variation period governed by the geometric configuration of the orbital plane relative to the Sun.

Components at approximately 133–150 h and 57–86 h correspond to 2/5 and 1/5 of the fundamental period, respectively. In the time–frequency spectra, these components manifest primarily as regular fluctuations of the main ridge amplitude rather than as consistently separable independent spectral lines, and are therefore more appropriately interpreted as envelope modulation time scales.

**Figure 7:**
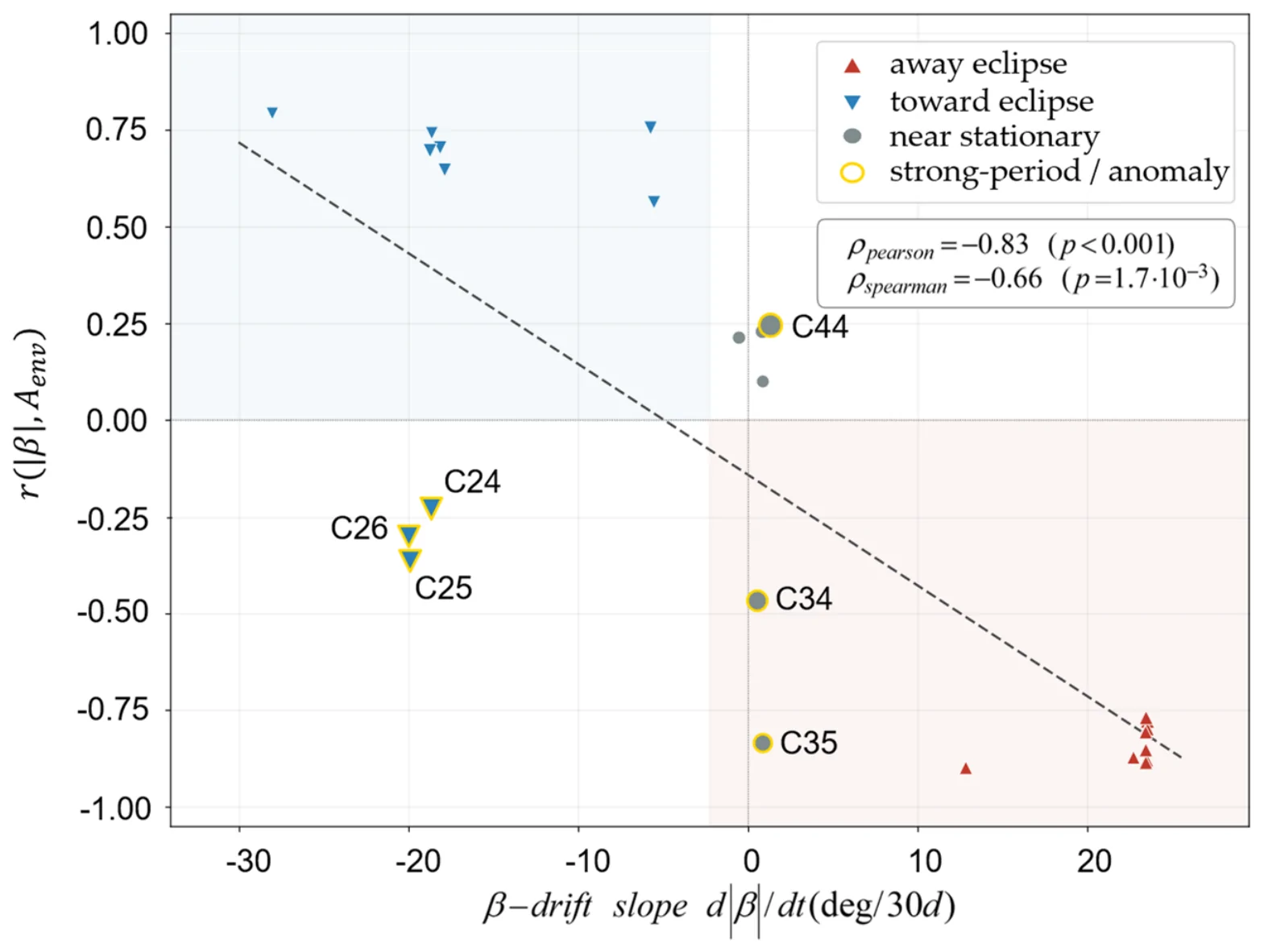
Relationship between β-angle drift slope and main ridge envelope correlation coefficient.

Figure 7 presents a statistical analysis of 26 satellites, with the 30-day β-angle drift slope as the horizontal axis and the main ridge correlation coefficient as the vertical axis. The results reveal a negative correlation between the two quantities, the β-angle drift slope and the envelope correlation coefficient are of opposite signs. Nineteen satellites fall into the second and fourth quadrants (one-sided binomial test significance probability excluding chance agreement).

The overall Pearson correlation coefficient is −0.83 (*p* < 0.001) and the Spearman rank correlation coefficient is −0.66 (*p* = 0.0017). The correlation may be influenced by a few outlier satellites or nonlinear factors, but the direction is consistent and statistical significance remains valid. Notably, excluding the anomalous satellite C35 further strengthens the correlation between the β-angle drift slope and the envelope correlation coefficient, thereby enhancing the credibility of the argument. Among the 10 satellites far from Earth’s shadow, the median correlation coefficient reaches −0.83, indicating strong statistical correlation. Taken together, the three components form a coupled hierarchy: the fundamental period acts as the carrier, the sub-harmonics appear as its fractional time scales, and the β-angle modulation governs their common amplitude envelope; the time-varying amplitude and phase therefore evolve coherently rather than independently.

### 3.3. Short-Term Prediction Validation

#### 3.3.1. Evaluation Metrics

DOY 212 is used as a single-day independent forecast set to conduct a comparative evaluation of five strategies: fixed baseline, FFT-anchored, KNN, RBF-KRR, and uREE. Per-satellite accuracy is quantified by the root mean square error:

(22) $$RMS_{s}=\sqrt{\frac{1}{N}{\sum_{i=1}^{N}\left[{\tilde{y}}_{s}(t_{i})-y_{s}(t_{i})\right]}^{2}}$$

where ${\tilde{y}}_{s}(t_{i})$ is the predicted CPD value for satellite s at epoch $t_{i}$, and $y_{s}(t_{i})$ is the corresponding true value.

The overall accuracy across satellites is computed as the root mean square average:

(23) $$RMS_{overall}=\sqrt{\frac{1}{S}\sum_{s=1}^{S}RMS_{s}^{2}}$$

To dissect the error sources, a bias–dispersion decomposition is applied to the forecast residuals. The systematic bias level is quantified by the mean bias error:

(24) $$MBE=\frac{1}{S}\sum_{i=1}^{S}\left[{\tilde{y}}_{s}(t_{i})-y_{s}(t_{i})\right]$$

The residual standard deviation characterizes the random fluctuation level:

(25) $$STD=\sqrt{\frac{1}{N}{\sum_{i=1}^{N}\left[{\tilde{y}}_{s}(t_{i})-y_{s}(t_{i})-MBE\right]}^{2}}$$

Using $\bar{RMS^{2}}=\bar{MBE^{2}}+\bar{STD^{2}}$, the systematic bias contribution is defined as:

(26) $$P_{MBE}=\frac{MBE^{2}}{RMS^{2}}\cdot 100\%$$

#### 3.3.2. Forecast Results and Accuracy Comparison

**Figure 8:**
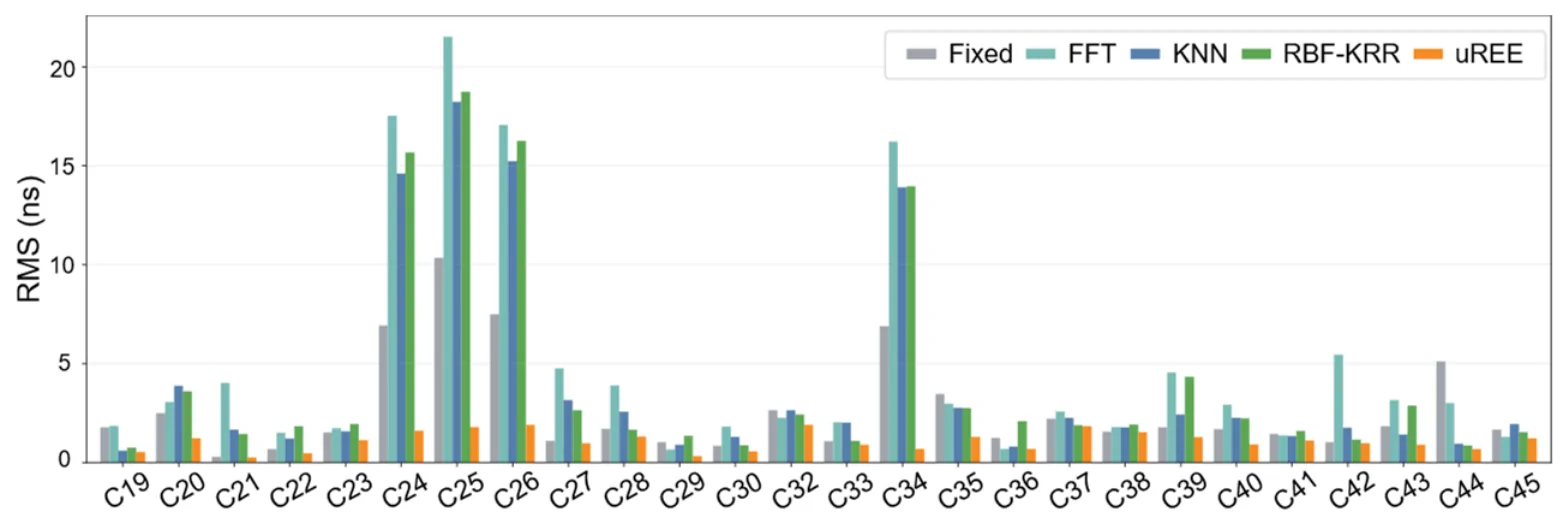
Per-satellite forecast residual RMS.

The per-satellite forecast RMS values for the five strategies on DOY 212 are shown in Figure 8. The uREE model achieves the best overall performance among the five strategies.

Per-satellite results show that uREE achieves significant improvement for satellites with strong periodicity. The RMS values of C24–C26, C34, and C44 are reduced to 2.023, 2.194, 3.200, 1.237, and 0.770 ns, respectively, representing improvements exceeding 57% relative to the fixed baseline. The FFT extrapolation, conversely, produces residual increases to 16–22 ns on strong periodicity satellites such as C24–C26. For weak structure satellites, including C21, C27, C38, and C42, the uREE automatically reverts to the fixed baseline accuracy through the adaptive mode’s contraction safeguard.

**Figure 9:**
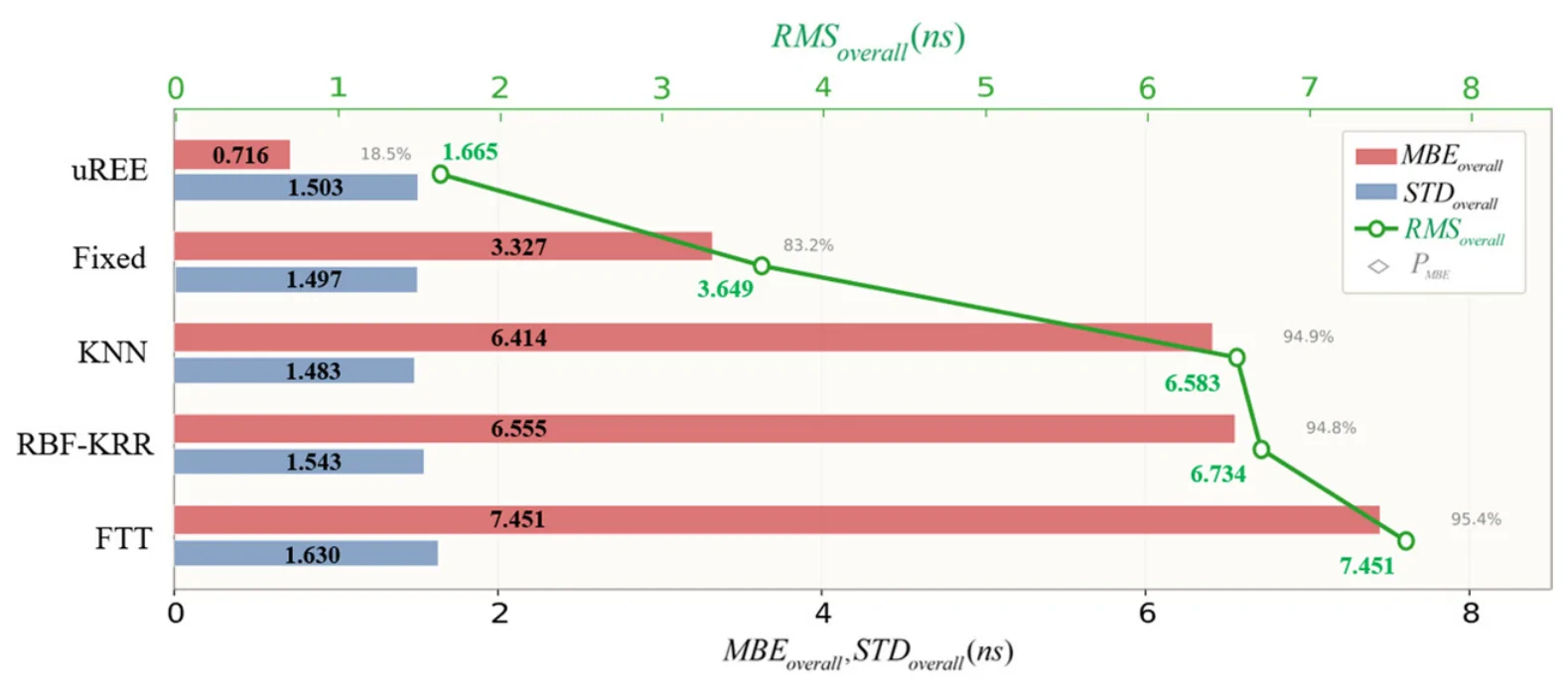
Bias–dispersion decomposition of overall forecast residuals.

The residual STD values of all five strategies are approximately 1.5 ns, whereas the MBE values differ substantially. This indicates that the forecast accuracy differences among the models are dominated entirely by systematic bias rather than random noise, underscoring the central role of bias modeling in CPD prediction. The MBE of uREE is only 0.716 ns, accounting for 18.5% of the total RMS, while the remaining four strategies all exceed 83% (Figure 9). uREE reduces the overall RMS from 3.649 ns (fixed baseline) to 1.665 ns, yielding a 54.4% accuracy improvement. Under identical test conditions, the overall RMS values of KNN, RBF-KRR, and FFT are 6.583, 6.734, and 7.627 ns, respectively, none of which outperforms the fixed baseline.

The degradation of the data-driven models below the fixed baseline is structural rather than configurational. The dominant CPD periods exceed all feature lag horizons and are incommensurate with the diurnal phase, so neither learner can perform phase-coherent continuation. Both models therefore revert toward the training mean with a wrong phase offset. The bias–dispersion decomposition confirms this: the STD stays at the common noise level (~1.5 ns), while the degradation appears entirely as systematic bias. All hyperparameters were tuned on the modeling set only, so the comparison is fair; the result exposes the bias accumulation failure mode that motivates the interpretable design of uREE.

## 4. Discussion

The three cross-satellite common periodic components demonstrate that the CPD possesses systematic time-varying structures. This finding prompts a shift of the research perspective from static bias estimation to time-varying periodic characterization.

The fundamental period coincides with 14 sidereal days, and the IGSO fundamental period (329–335 h) far exceeds twice the orbital repeat period (47.6 h), ruling out individual orbital mechanics as the driver [29]. This component most likely originates from Earth rotation-related systematic effects, such as periodic variations in ground tracking network geometry or residual errors in Earth orientation parameter models. Regarding the sub-harmonics, the absence of a 48 h orbital component in IGSO satellites provides key evidence for ruling out an orbital harmonic interpretation, supporting the fractional harmonic origin of the fundamental period.

The envelope modulation components are negatively correlated with the β-angle drift direction. This finding is consistent with previously reported β-angle- and eclipse season-related variations in satellite clock periodicity: Senior et al. [13] observed amplitude modulation by eclipse season in GPS clock offsets, and Zhao et al. [18] reported qualitatively that the spectral power of the dominant BDS periodic term is linked to the β-angle. The present study extends these findings to the CPD level by quantifying the statistical relationship between the envelope modulation and the β-angle drift direction.

The incremental contribution of this study over prior work is three-fold. Senior et al. [13], Zhao et al. [18], and Chen et al. [30,31] discovered periodic phenomena of satellite clock offsets. In contrast, the present study targets the CPD and quantifies the joint time-varying evolution of its amplitude and phase. The STFT-based model of Zhao et al. [18] compensates periodic terms under a fixed window, whereas uREE extrapolates envelope trend and phase evolution through satellite adaptive modes without window-length selection. The bias–dispersion decomposition further attributes the forecast gain to systematic bias mitigation. The research perspective is thus extended from periodicity discovery of clock offsets to time-varying characterization and physically interpretable forecasting of clock product deviations.

The accuracy advantage of uREE stems from the mitigation of systematic bias (18.5% contribution) rather than random noise suppression, with comparable STD levels across all five strategies. This result explains why data-driven methods (KNN, RBF-KRR), despite their proficiency in nonlinear mapping, fail to eliminate the systematic bias introduced by nonstationary periodic structures.

The proposed framework is transferable to resource-constrained platforms. The uREE forecast depends only on envelope trend and phase evolution, so its evaluation introduces negligible computational overhead, whereas the NTFT modeling can be performed centrally by augmentation providers. For smartphones and other consumer-grade GNSS terminals, uncorrected CPD propagates into ranging measurements and biases positioning and timing. An a priori periodic correction thus improves real-time accuracy at minimal cost. A representative scenario is GNSS positioning aided by pedestrian dead reckoning (PDR) in urban areas. During GNSS outages, PDR maintains short-term relative displacement. Once signals are reacquired, the a priori CPD correction accelerates filter re-convergence. The method is likewise applicable to intelligent transportation, the Internet of Things, and wearable devices.

The current validation has several limitations. The forecast is limited to a single day (DOY 212), and the 30-day modeling window covers a restricted β-angle range. The robustness of uREE under extreme conditions such as the eclipse season remains unverified. Deep-learning baselines are not included, as the per-satellite 30-day arcs provide limited training samples and the bias–dispersion analysis indicates that accuracy differences arise from systematic bias rather than learning capacity. Several directions merit further investigation. The universality of the envelope modulation law should be verified using multi-season and complete eclipse cycle β-angle datasets. The forecast horizon should be extended to 7–14 days to evaluate long-timescale error accumulation. Solar radiation pressure prior information can be incorporated to constrain the periodic modeling and enhance physical interpretability. Comparative evaluation against deep learning baselines on extended multi-season datasets will further substantiate the advantage of the interpretable structure.

## 5. Conclusions

This study establishes a framework based on the NTFT dynamic ridge for nonstationary periodic structure identification and uREE short-term forecasting of CPD. The main conclusions are as follows:

The CPD contains three cross-satellite clustered periodic components (fundamental period, sub-harmonics, and envelope modulation). The NTFT reduces the modeling residual STD from 5.273 ns to 0.429 ns.

The β-angle modulates the time-varying direction of the envelope rather than the absolute amplitude. The envelope correlation coefficient is negatively correlated with the β-angle drift direction (Pearson r = −0.83, *p* < 0.001).

The uREE reduces the forecast RMS from 3.649 ns to 1.665 ns (54.4% improvement), with the systematic bias contribution decreasing to 18.5%. For satellites with strong periodicity, the improvement exceeds 57%; for weak structure satellites, the adaptive mode automatically reverts to the fixed baseline.

The NTFT dynamic ridge provides a preset-free analytical approach for nonstationary periodic identification of CPD. The NTFT dynamic ridge provides a preset-free analytical approach for nonstationary periodic identification of CPD. Meanwhile, uREE provides a methodological framework for physically interpretable forecasting of real-time clock bias.

## Figures and Tables

**Table 1:** Comparison of the proposed framework with counterpart methods.

Method	Stationarity Assumption	Period Prior	Physical Interpretability	Forecast
FFT	Strong: fixed harmonics over the whole span	Fixed harmonic periods	Partial: constant amplitude and phase	No
STFT-TFAM	Weak: localized spectra	Window length	Partial: dominant-term power tracking	Yes, via periodic compensation
EMD	Weak	No	Low: modes lack physical constraint	No
VMD	Weak	Preset mode number	Low: mode number chosen empirically	No
EWT-LSTM	Weak	No	None: black-box mapping	Yes via LSTM
NTFT-uREE	Nonstationary allowed	No periodogram screening	Yes: time-varying A, f, and φ trajectories	Yes via uREE

*Note: A, f, and φ denote amplitude, frequency, and phase, respectively*.

**Table 2:** Periodic structure of CPD series.

Sat	Dom.P	Dom.A	Subh.P	Subh.A	Env.P	Env.A	Sat	Dom.P	Dom.A	Subh.P	Subh.A	Env.P	Env.A
C19	335.2	2.69	135.1	0.85	61.6	0.71	C33	334.0	2.57	136.9	0.80	38.2	0.64
C20	338.6	2.43	141.6	0.78	66.5	0.81	C34	338.1	5.95	66.4	1.10	38.6	0.78
C21	330.6	2.83	136.9	0.63	63.3	0.69	C35	258.6	4.84	72.4	1.17	40.4	0.97
C22	335.4	2.92	62.6	0.71	33.0	0.57	C36	332.4	2.72	28.0	0.93	133.2	0.53
C23	337.9	2.38	60.8	0.76	25.6	0.66	C37	332.8	2.61	137.6	0.60	62.9	0.73
C24	335.7	6.62	61.9	0.98	39.1	0.83	C38	331.1	2.53	139.7	0.72	23.9	0.83
C25	335.3	6.76	65.3	1.07	41.1	0.82	C39	335.1	2.12	134.6	0.98	59.0	1.05
C26	343.0	6.63	63.8	1.05	38.8	0.85	C40	329.4	2.53	138.0	0.74	24.0	0.97
C27	337.4	2.75	66.4	0.83	28.8	0.76	C41	335.7	2.67	62.0	0.81	27.3	0.67
C28	338.0	2.64	140.9	0.78	61.1	0.79	C42	337.4	2.83	61.6	0.82	107.8	0.61
C29	335.5	2.39	25.0	0.69	137.5	0.67	C43	330.6	2.90	138.4	0.68	61.5	0.60
C30	330.5	2.60	134.9	0.57	86.0	0.43	C44	339.7	7.26	140.2	1.77	73.5	1.42
C32	325.7	2.67	145.6	0.73	57.9	0.84	C45	339.9	2.76	149.7	0.68	83.4	0.75

*Note: Sat = satellite PRN; Dom. = dominant; Subh. = sub-harmonic; Env. = envelope modulation; P = period (h); A = amplitude (ns)*.

## Data Availability

The data used in this study are not publicly available but may be obtained from the corresponding author upon reasonable request. The modeling dataset consists of BDS-3 PPP-B2b real-time clock products spanning DOY 182–211 of 2025, with CODE post-processed precise clock products as the reference, and DOY 212 is reserved as an independent prediction set. All CPD sequences are uniformly resampled at a 5 min interval.

## Abbreviations

The following abbreviations are used in this manuscript:

BDS-3	BeiDou-3 Navigation Satellite System
GPS	Global Positioning System
GEO	Geostationary Earth Orbit
IGSO	Inclined Geosynchronous Orbit
MEO	Medium Earth Orbit
CPD	Clock Product Deviation
CODE	Center for Orbit Determination in Europe
NTFT	Normal Time–Frequency Transform
FFT	Fast Fourier Transform
CWT	Continuous Wavelet Transform
EMD	Empirical Mode Decomposition
VMD	Variational Mode Decomposition
uREE	unified Ridge Envelope Extrapolation
DOY	Day of Year
STD	Standard Deviation
RMS	Root Mean Square
MBE	Mean Bias Error
KNN	K-Nearest Neighbors
RBF-KRR	Radial Basis Function Kernel Ridge Regression
IRLS	Iteratively Reweighted Least Squares
MAD	Median Absolute Deviation
CV	Coefficient of Variation
TGD	Timing Group Delay
SRP	Solar Radiation Pressure
